# MEK inhibition induced downregulation of MRP1 and MRP3 expression in experimental hepatocellular carcinoma

**DOI:** 10.1186/1475-2867-13-3

**Published:** 2013-01-15

**Authors:** Shibo Lin, Katrin Hoffmann, Zhi Xiao, Nan Jin, Uwe Galli, Elvira Mohr, Markus W Büchler, Peter Schemmer

**Affiliations:** 1 Department of General and Transplant Surgery, Ruprecht-Karls-University, Im Neuenheimer Feld 110, Heidelberg, 69120, Germany; 2 Department of Breast Surgery, Xiangya Hospital, Zhongnan University, Changsha, 410008, China; 3 Department of Hematology, Oncology, and Rheumatology, Ruprecht-Karls-University, Heidelberg, 69120, Germany

**Keywords:** Hepatocellular carcinoma, MEK, MRP1, MRP3, Multidrug resistance

## Abstract

**Background:**

Hepatocellular carcinoma (HCC) exhibits strong intrinsic and acquired drug resistance which is the main obstacle to chemotherapy. Overexpression of ATP binding cassette (ABC) proteins correlates with activation of mitogen activated protein kinase (MAPK) pathway in HCC. Here, we systematically investigated the inhibition of MAPK pathway and its role in regulating HCC cell growth as well as ABC proteins MRP1 and MRP3 expression.

**Methods:**

The Raf1 kinase inhibitor (GW5074) and different MEK inhibitors (U0126 and AZD6244) were used to treat HCC cells to identify their effects on HCC cell growth and ABC proteins expression *in vitro*. Cell viability tests were performed after the treatment of MAPK pathway inhibitors and in combination with gemcitabine or doxorubicin. Western blot was applied to assess the changes of MAPK pathway and protein expression of MRP1 and MRP3. Flow cytometry was used to measure intracellular doxorubicin accumulation after the treatment of MEK inhibitors.

**Results:**

Both Raf1 inhibitor (GW5074) and MEK inhibitors (U0126 and AZD6244) suppressed HCC cell growth in a dose dependent manner. Pre-treatment of MEK inhibitor U0126 or AZD6244 sensitized HCC cells to gemcitabine or doxorubicin based chemotherapy. Raf1 inhibitor GW5074 had no effect on MRP1 and MRP3 protein expression. Treatment of gemcitabine or doxorubicin activated phosphorylated ERK and induced the upregulation of MRP1 and MRP3. MEK inhibitors U0126 and AZD6244 deactivated phosphorylated ERK, decreased endogenous MRP1 expression, reversed gemcitabine or doxorubicin induced MRP1 and MRP3 upregulation, and increased the intracellular doxorubicin accumulation.

**Conclusion:**

This study provides evidence that MEK inhibitors sensitize HCC cells to chemotherapy by increasing intracellular chemodrug accumulation. MEK inhibirors U0126 and AZD6244 reduced MRP1 as well as MRP3 expression, and may contribute partially to the sensitization. The combination of MEK inhibitor and conventional chemotherapy may offer new therapeutic option for the treatment of resistant HCC.

## Background

Hepatocellular carcinoma (HCC) is the third most common cause of cancer mortality and causes more than half a million deaths annually worldwide [1,2]. The number of new cases of primary liver cancer increases globally and HCC accounts for 70% to 85% of them [3]. Potentially curative treatment, including liver resection, transplantation and local ablation, could provide promising 5-year-survival rate up to 75% [4], however, less than 20% of HCC patients are eligible for these treatment [5]. For patients who have either recurrent disease after surgical therapy or initially advanced HCC, sorafenib is considered to be the first-line treatment [6]. However, the response to sorafenib treatment is still low (2%) [7,8]. Furthermore, chemotherapeutic options for HCC are limited. Systemic chemotherapy with doxorubicin, gemcitabine or combined regiments for palliative strategy was reported to provide only marginal effect on survival of HCC patients [9-11]. A high intrinsic and acquired drug resistance in HCC is mainly responsible for this failure of the systemic chemotherapy [12].

The mechanisms of drug resistance in tumour cells are heterogeneous, including increased efflux of anticancer agents by ABC proteins, blocked apoptosis, activated DNA repair and enhanced detoxifying systems [13]. Among them, ABC proteins contribute to the major form of drug resistance by increasing the efflux of anticancer drugs out of cancer cells [14]. Our previous analysis revealed that, among these ABC proteins, MRP1 and MRP3 were overexpressed in HCC tissue and may contribute to the high intrinsic drug resistance [15]. We also previously demonstrated that the phenotype of acquired drug resistance could be induced by conventional anticancer agents in HCC cells. Treatment of gemcitabine and doxorubicin to HCC cells resulted in an upregulation of MRP1 and MRP3 gene and protein expression [16]. Thus, inhibition of MRP1 and MRP3 might reverse multidrug resistance and improve chemotherapeutic efficiency in HCC.

Overexpression and abnormal activation of the MAPK pathway were previously detected and correlated statistically with MRP1 overexpression in HCC tissue [15,17-19]. ERK activation induced by chemotherapy was observed in HCC cells [16]. Furthermore, Zhang et al. shown that the basal level of the phosphorylated ERK in HCC cells affected their chemosensitivity to 5-fluorouracil treatment [20]. These results suggested that MAPK pathway and drug resistance may interact with each other in HCC. Modulation of ABC proteins expression with tyrosine kinase inhibitors was proven to be feasible. In HCC, Hoffmann et al. reported that both gefitinib and sorafenib decreased gemcitabine and doxorubicin induced upregulation of ABC proteins and restored the chemosensitivity [16,21]. These reversal effects originated from inhibition at the receptor level of the tyrosine kinase pathway. However, the involvement of the downstream MAPK pathway, such as Raf1 and MEK, in mediating the ABC proteins expression remains unclear in HCC.

The objective of this investigation was to elucidate the interaction between two key kinases within the MAPK pathway (Raf1 and MEK) and ABC proteins expression in HCC. Highly selective inhibitors which inhibited the Raf1 kinase (GW5074) and the MEK activity (U0126 and AZD6244) were applied to identify their effects on the MRP1 and MRP3 protein expression.

## Results

### GW5074 inhibited HCC cell growth and Raf1 expression

**Figure 1:**
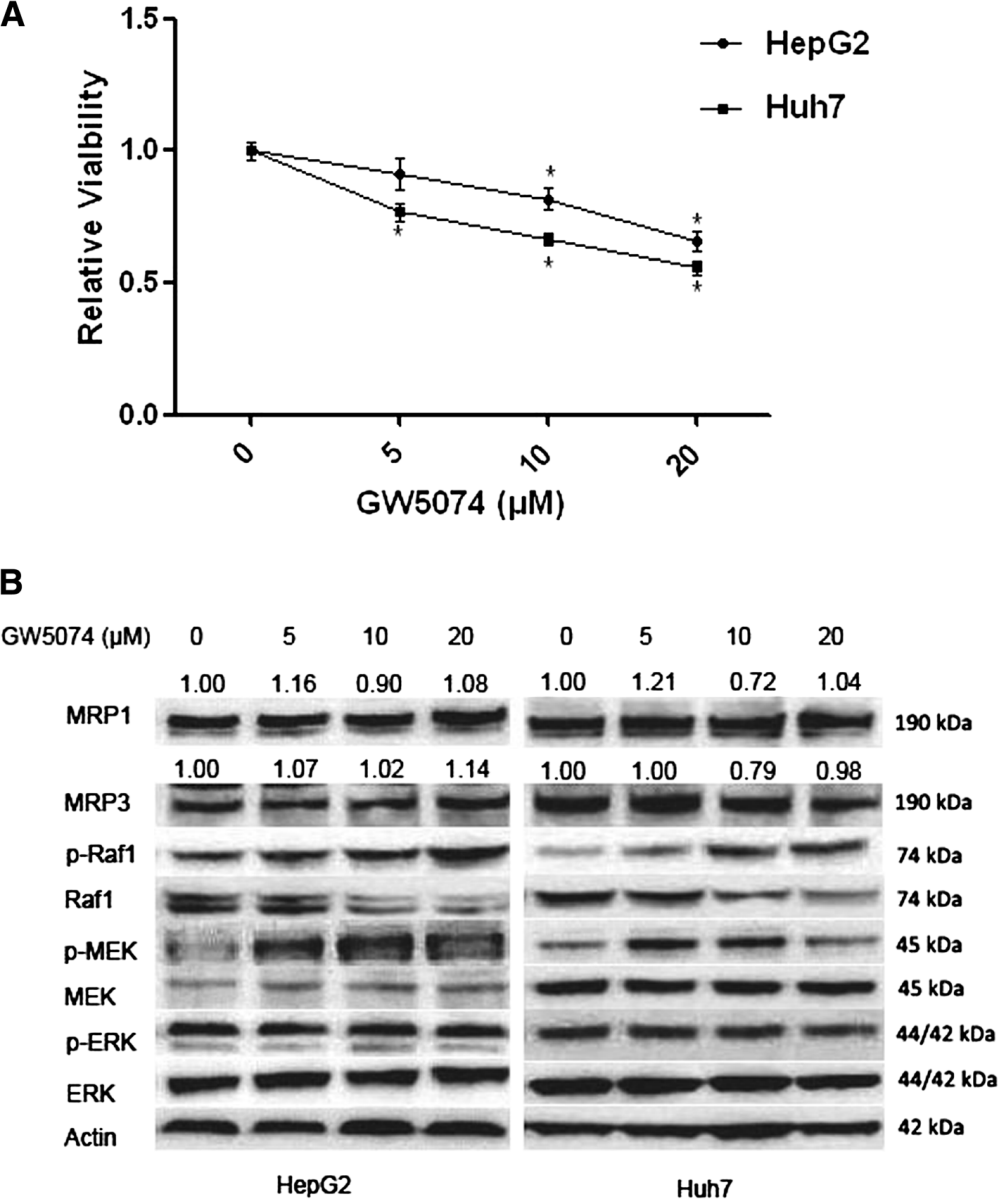
**Role of GW5074 on HCC cell growth and ABC proteins expression. A**: HepG2 and Huh7 were treated with three different concentrations (5 μM, 10 μM and 20 μM) of Raf1 kinase inhibitor GW5074. After 48 hours treatment, cell viability test was performed to measure the relative viability. * *P*<0.05 compared with control group. **B**: HepG2 and Huh7 cells were treated with three different concentrations (5 μM, 10 μM and 20 μM) of GW5074 for 48 hours, and then cells were lysed and western blot was performed.

To determine the role of Raf1 inhibition on HCC cell growth and drug resistance, HCC cells were treated with the Raf1 kinase inhibitor GW5074 (5 μM–20 μM). GW5074 exhibited a dose-dependent cell growth inhibition in HepG2 and Huh7 cells (Figure 1A). We further examined the effects of GW5074 on MAPK pathway and protein expression of MRP1 and MRP3 in HCC cells. Western blot analysis revealed that GW5074 dose-dependently downregulated Raf1 but also increased phosphorylation of Raf1 (Figure 1B). GW5074 activated p-MEK at the concentration of 5 μM, but the activation declined as the concentration increased. Furthermore, we showed that GW5074 had no effect on MRP1 and MRP3 protein expression in both HCC cell lines (Figure 1B). As shown in Figure 1B, Raf1 inhibition by GW5074 did not exert an inhibitory effect on p-MEK and p-ERK, but activate the p-MEK. It was reported that heterodimerization of B-Raf with Raf1 induced by Raf kinase inhibitor GW5074 contributed to the activation of the downstream MAPK signalling in cells with mutant k-ras or wild-type B-Raf, such as HepG2 [22,23]. This result indicated Raf1 as the first downstream of the MAPK pathway is involved in mediating HCC cell growth, but plays no significant role in the regulation of MRP1 and MRP3 expression. Thus, it was of interest to know whether downstream of the Raf1 kinase pathway, such as MEK or ERK, was involved in mediating MRP1 and MRP3 expression.

### MEK inhibitors inhibited HCC cell growth and enhanced chemosensitivity

**Figure 2:**
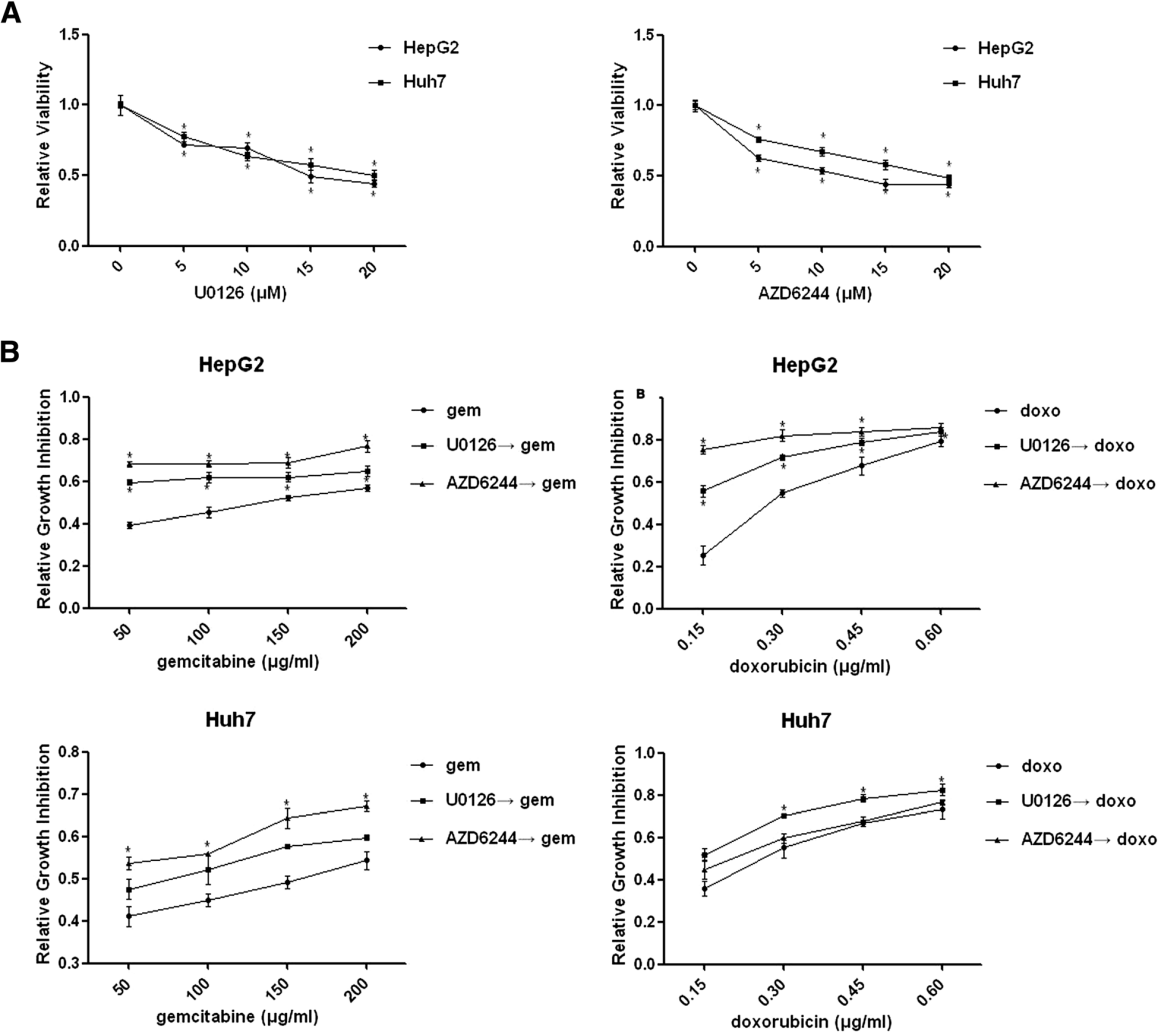
**AZD6244 inhibited HCC cell growth and enhanced chemosensitivity. A**: HepG2 and Huh7 were treated with four different concentrations (5 μM, 10 μM, 15 μM and 20 μM) of MEK inhibitor U0126 and AZD6244. After 48 hours treatment, cell viability test was performed to measure the relative viability. * *P*<0.05 compared with control group. **B**: HepG2 and Huh7 cells were treated with U0126 (10 μM) and AZD6244 (10 μM) for 24 hours, followed by gemcitabine (50 μg/ml, 100 μg/ml, 150 μg/ml and 200 μg/ml) or doxorubicin (0.15 μg/ml, 0.30 μg/ml, 0.45 μg/ml and 0.6 μg/ml) for another 48 hours. Cell viability test was performed to measure the relative viability. *: synergistic effect was detected.

To determine whether MEK inhibition could influence HCC cell growth, HCC cells were treated with the MEK inhibitor U0126 (5 μm–20 μM) or AZD6244 (5 μm–20 μM) for 48 hours. Both U0126 and AZD6244 exerted dose-dependent inhibition on HepG2 and Huh7 cell growth (Figure 2A). These results indicated that downstream of MAPK pathway was involved in regulating HCC cell growth.

We next investigated whether MEK inhibitors could enhance chemotherapeutic effects. HCC cells were pretreated with U0126 (10 μM) or AZD6244 (10 μM) for 24 hours, followed by different concentrations of gemcitabine or doxorubicin for another 48 hours. As shown in Figure 2B, the pretreatment of U0126 and AZD6244 synergistically sensitized HepG2 cells to gemcitabine and doxorubicin induced growth inhibition. U0126 also synergistically enhanced the chemosensitivity of doxorubicin in Huh7 cells. Similar synergistic effect of growth inhibition was observed when Huh7 cells were pretreated with AZD6244 followed by gemcitabine. However, U0126 did not exert synergistic effect on gemcitabine induced Huh7 cell growth inhibition. And AZD6244 did not sensitize the chemotherapeutic effect of doxorubicin in Huh7 cells, either.

### MEK inhibitors reversed MRP1 and MRP3 expression

**Figure 3:**
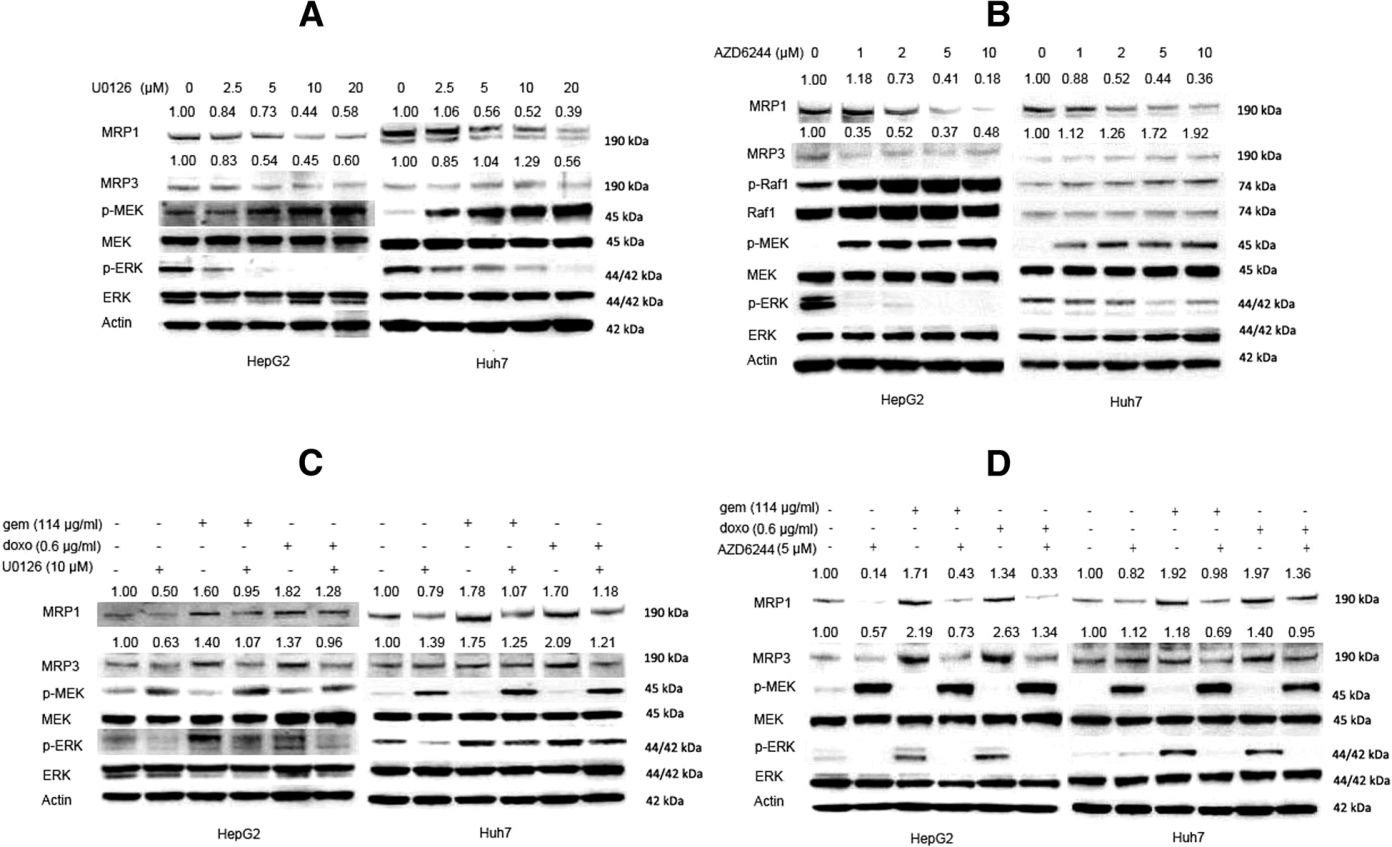
**MEK inhibitors reversed MRP1 and MRP3 expression. A**: HepG2 and Huh7 were treated with four different concentrations (2.5 μM, 5 μM, 10 μM and 20 μM) of U0126. After 48 hours of U0126 treatment, the cells were lysed and western blot was performed. **B**: HepG2 and Huh7 were treated with four different concentrations (1 μM, 2 μM, 5 μM and 10 μM) of AZD6244. After 48 hours treatment, the cells were lysed and western blot was performed. **C**: HepG2 and Huh7 were treated with gemcitabine (114 μg/ml) or doxorubicin (0.6 μg/ml) for 48 hours, and followed by another 24 hours of U0126 (10 μM) treatment. Then the cells were lysed and western blot was performed. **D**: HepG2 and Huh7 were treated with gemcitabine (114 μg/ml) or doxorubicin (0.6 μg/ml) for 48 hours, and followed by another 48 hours of AZD6244 (5 μM) treatment. Then cells were lysed and western blot was performed.

Western blot analysis revealed that MEK inhibitors U0126 and AZD6244 modulated the MAPK pathway by increasing the p-MEK levels and decreasing the p-ERK levels. An inhibition of endogenous MRP1 expression was observed in a dose-dependent manner after 48 hours of U0126 or AZD6244 treatment (Figure 3A and 3B). Both U0126 and AZD6244 exerted downregulatory effect on endogenous MRP3 expression in HepG2 cells. U0126 decreased MRP3 expression at the concentration of 20 μM; however, AZD6244 dose-dependently increased MRP3 expression in Huh7 cells.

We next examined whether MEK inhibitors had similar effects on chemotherapy induced upregulation of MRP1 and MRP3. HCC cells were exposed to gemcitabine or doxorubicin for 48 hours, followed by U0126 or AZD6244 for another 24 hours. Activation of the MAPK pathway (increased p-ERK) and an upregulation of MRP1 and MRP3 protein were observed after doxorubicin or gemcitabine treatment in both cell lines (Figure 3C and 3D). However, MEK inhibitors U0126 and AZD6244 reversed the upregulation of p-ERK as well as MRP1 and MRP3. These results suggested that MEK kinase was involved in regulating endogenous as well as chemotherapy induced MRP1 and MRP3 protein expression in HCC cell lines.

### U0126 and AZD 6244 increased intracellular doxorubicin accumulation

**Figure 4:**
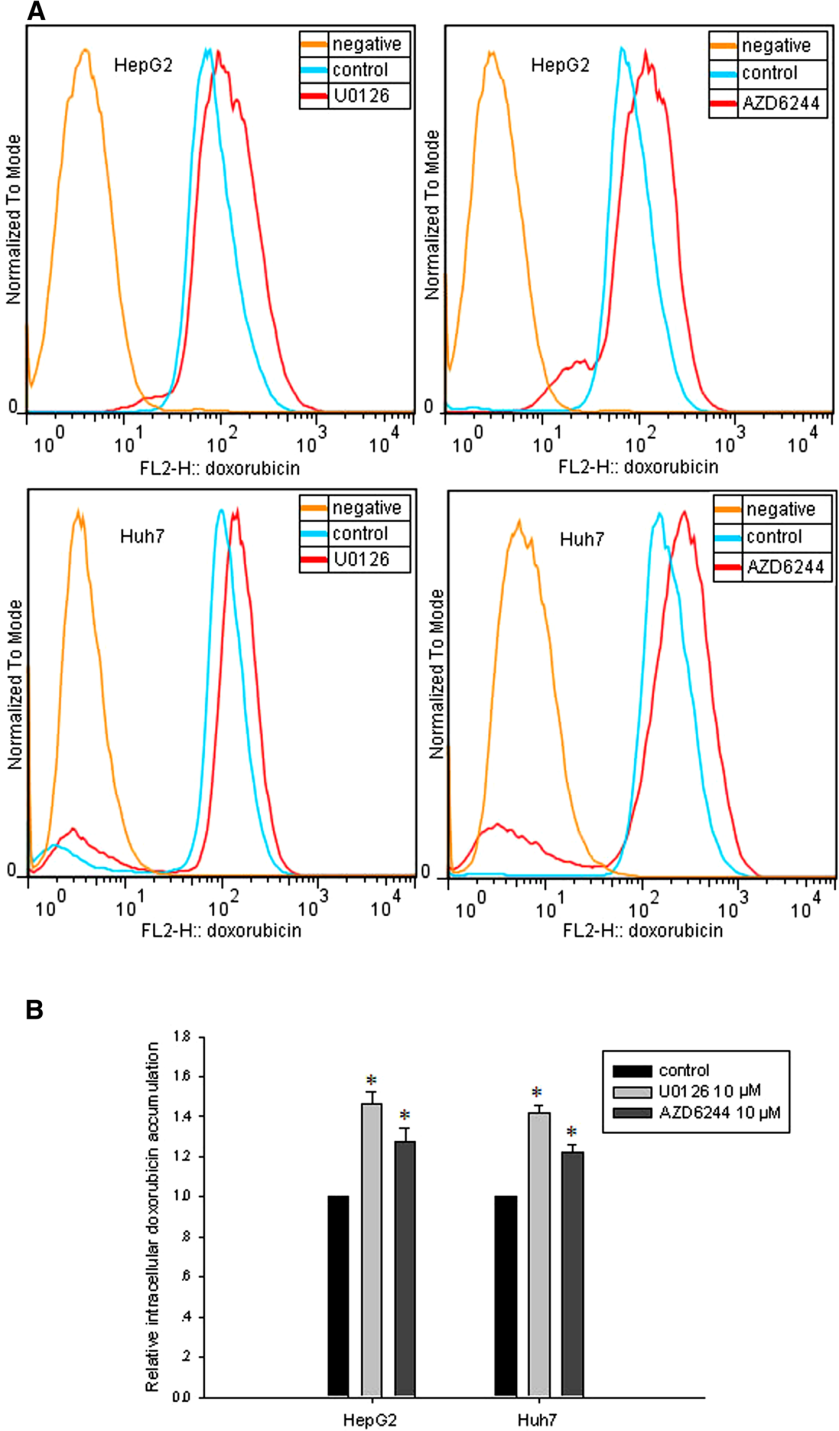
**U0126 and AZD6244 increased intracellular doxorubicin accumulation. A**: HepG2 or Huh7 cells were treated with U0126 or AZD6244 for 48 hours, followed by incubation of doxorubicin (6 μg/ml) for 2 hours. Then the cells were trypsinized and resuspended in PBS followed by FACS analysis. **B**: Statistical analysis of FACS results. * *P<0.05* compared with control group.

Based on enhanced chemosensivity to doxorubicin and decreased MRP1 expression induced by MEK inhibitors in HepG2 cells, we hypothesized that MEK inhibitors might increase intracellular accumulation of doxorubicin by decreasing ABC proteins efflux ability. To confirm this, FACS analysis was performed to measure doxorubicin accumulation after U0126 or AZD6244 treatment (Figure 4A). In HepG2 cells, we observed that the density of intracellular doxorubicin fluoresces increased by 46.5% after U0126 treatment and 42.0% after AZD6244 treatment (Figure 4B). In Huh7 cells, U0126 and AZD6244 treatment exerted 27.4% and 21.8% increase of intracellular doxorubicin accumulation, respectively (Figure 4B). These results suggested that MEK inhibitors increased intracellular accumulation of chemodrug.

## Discussion

Hepatocellular carcinoma exhibits its high intrinsic multidrug resistance (MDR) phenotype through overexpression of MRP1 and MRP3, which hampers successful chemotherapeutic treatment [24,25]. Thus, modulation of these overexpressed ABC proteins may diversify the therapeutic choices for HCC. In present study, we investigated the effects of downstream MAPK pathway (Raf1 and MEK) inhibition on chemosensitivity as well as MRP1 and MRP3 expression in HCC. We demonstrated that MEK inhibition sensitized HCC cells to gemcitabine and doxorubicin. And we further indicated that downregulation of MRP1 and MRP3 by MEK inhibitors might contribute partially to this sensitization.

Sustained cell proliferation is one of the main features of cancer [26] and MAPK pathway is involved in regulating cell proliferation [27]. Raf1 or MEK inhibitor was reported to suppress HCC cells growth [28-30]. Furthermore, combination of MEK inhibitor (AZD6244) and doxorubicin lead to synergistic HCC tumor growth inhibition in mouse models [31]. In line with previous investigations, our data showed that monotherapy of either Raf1 inhibitor (GW5074) or MEK inhibitors (U0126 and AZD6244) exhibited a dose-dependent growth inhibition of HCC cells. Furthermore, we observed that pretreatment of MEK inhibitors sensitized HCC cells to doxorubicin or gemcitabine, and increased intracellular doxorubicin accumulation. Based on these results, we hypothesized that this additional cell growth inhibition might originate from increased accumulation of chemotherapeutic reagents in cancer cells. AZD6244, also known as Selumetinib or ARRY-142886, has already been tested in phase II clinical trial for hepatocellular carcinoma which indicated that AZD6244 had minimal single-agent activity despite evidence of suppression of target activation [32]. Our results suggested that combination of AZD6244 with conventional anticancer drugs may be an optional therapeutic choice.

The aim for the modulation of ABC proteins is to improve the efficacy of anticancer drugs through increasing intracellular anticancer drug accumulation [33]. Abundant evidence has shown that tyrosine kinase inhibitors (TKIs) could modulate ABC proteins either in function or in mRNA and protein level. Dohse et al. reported that imatinib and dasatinib, which inhibit BCR-ABL tyrosine kinase, could overcome ABCG1 and ABCG2 transporting function [34]. Similar results were obtained from vandetanib (VEGFR and EGFR inhibitor) through functional inhibition of ABCB1, ABCC1 and ABCG2 [35,36]. And U0126 (MEK inhibitor) promoted PGP protein degradation in colorectal cancer was also reported [35]. Previous studies in our group indicated that gefitinib (EGFR inhibitor) and sorafenib (VEGFR, PDGFR-h and Raf inhibitor) exerted inhibitory effects on mRNA expression of ABCB1, ABCC1, ABCC2 and ABCC3 [16,21]. Our current results indicated that MEK inhibitors decreased the endogenous MRP1 protein expression, which contributed to intrinsic drug resistance in HCC [25]. As previously reported, acquired drug resistance could be induced by short time chemotherapy, but last for more than 6 weeks [37]. In HCC, conventional chemotherapy enabled cancer cells to acquire drug resistance through overexpression of MRP1 and MRP3. However, MEK inhibitors significantly reversed the upregulation of MRP1 and MRP3 induced by gemcitabine and doxorubicin. Based on these data, we speculate that MEK inhibitors might reverse both intrinsic and acquired drug resistance in HCC cells through inhibition of MRP1 and MRP3 protein expression.

In contrast to the down-regulation of MRP1 and MRP3 protein expression, mRNA expression was increased after the U0126 treatment, especially for MRP3 (data not shown). Furthermore, U0126 also exerted an enhancive effect on ABCC3 mRNA upregulation induced by gemcitabine and doxorubicin, while MRP3 protein expression was decreased after U0126 treatment. Dreuw et al. also reported similar results, namely that exposure of U0126 to dermal fibroblasts enhanced ABCC3 mRNA expression [38]. The post transcriptional regulation may well be responsible for this phenomenon. By using pulse chase experiments, Katayama et al. reported that U0126 promoted PGP degradation but did not affect its biosynthesis [35]. Moreover, it was reported that MEK inhibitor could induce transcriptional upregulation of endogenous BCRP through the inhibition of the MEK-ERK-RSK pathway, but promote post-transcriptional protein degradation of endogenous BCRP through the inhibition of the MEK-ERK-non-RSK pathway in breast cancer cells [39]. Further experiments indicated that the 5’ end of the ABCB1 mRNA in normal colon cancer cells was shorter than in doxorubicin resistant breast cancer cells, and alternative promoters were responsible for the PGP post-transcriptional regulation, which exhibited increased ABCB1 mRNA expression but unchanged protein expression and PGP efflux function [40]. However, the mechanisms involved in post-transcriptional degradation of MRP1 and MRP3 require further elucidation.

MEK inhibitor exerted stronger downregulatory effect on the endogenous MRP1 expression than MRP3. The MRP1 expression is very low or even could not be detected in healthy human hepatocytes [41]. Significant inhibition of MRP1 expression and unchanged endogenous MRP3 expression would not result in severe physiological disorders of hepatocytes. This difference may be of great importance especially to the HCC patients with decompensated liver function who would usually get no therapy.

**Figure 5:**
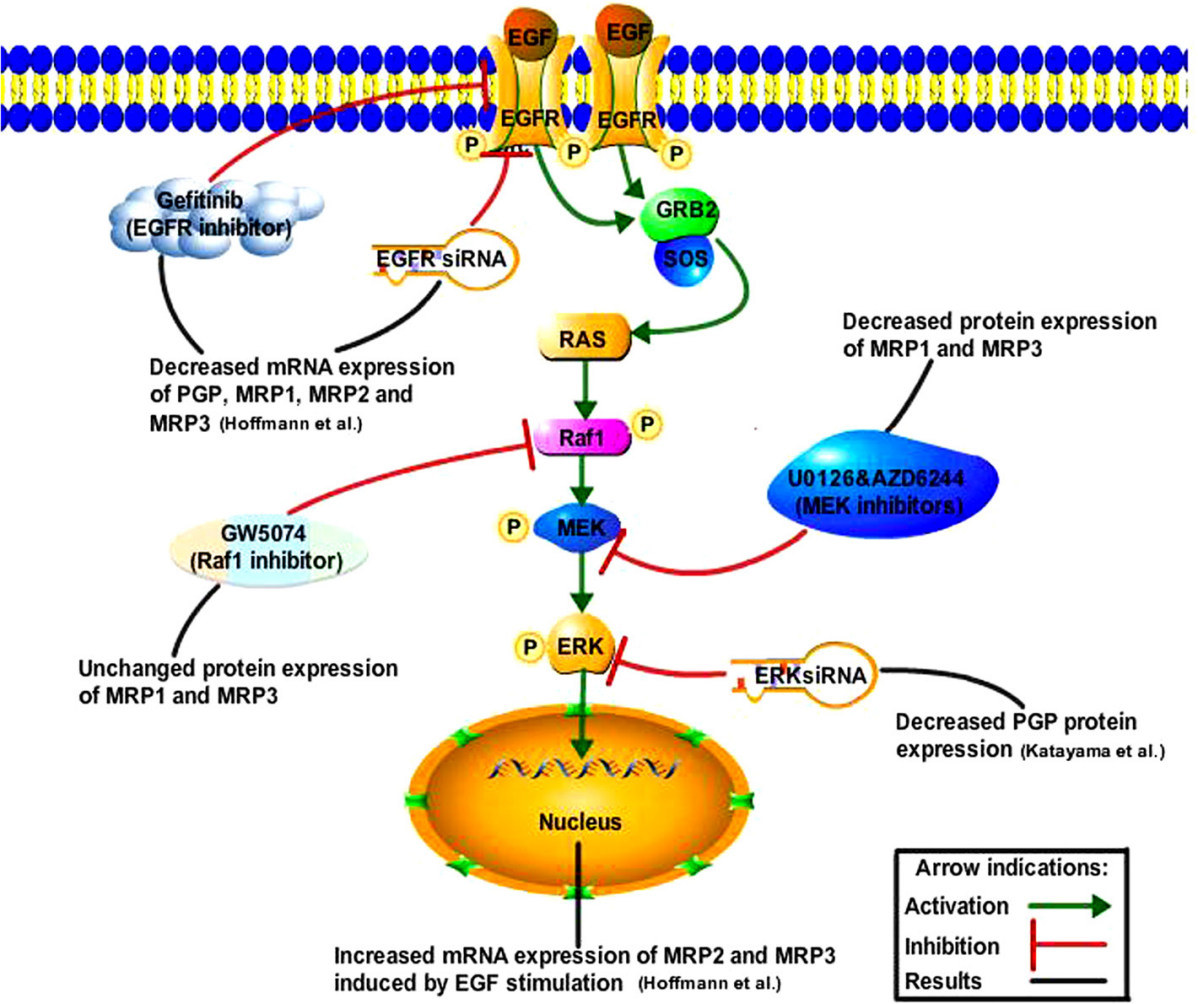
Hypothesized mechanism of involvement of the EGF pathway in the regulation of ABC protein expression.

Extensive evidence has shown that the EGF-Ras-MAPK pathway was involved in the regulation of ABC protein expression. EGF stimulation activated MAPK pathway, furthermore, enhanced the PGP expression, and promoted the ABCC1, ABCC2 as well as ABCC3 gene expression [16,42-44]. We previously reported that EGFR inhibition suppressed ABCB1, ABCC1, ABCC2 and ABCC3 mRNA expression [16]. Moreover, ERK siRNA decreased PGP expression was also demonstrated [35]. Here, we identified that downstream of the EGF pathway, MEK might be another target for reversing MRP1 and MRP3 expression. Based on these results, we hypothesized the involvement of the EGF pathway in the regulation of ABC protein expression as shown in Figure 5.

## Conclusions

Our results provide the evidence that the MAPK pathway is not only involved in the regulation of HCC cell proliferation but also may be involved in the regulation of multidrug resistance. For the first time, we systemically revealed that the inhibition of MEK with the MEK inhibitors U0126 or AZD6244 could lead to a downregulation of MRP1 and MRP3 expression in HCC *in vitro*. MEK inhibition might be a novel therapeutic option to reverse multidrug resistance in HCC patients.

## Materials and methods

### Cell lines and materials

Two human hepatocellular carcinoma cell lines (HepG2 and Huh7) were used for the *in vitro* experiments: HepG2 was purchased from Toni Lindl GmbH (Munich, Germany); Huh7 was a gift from Prof. Herr (Division of Molecular OncoSurgery, Ruprecht-Karls-University, Heidelberg, Germany). HepG2 cells were cultured in RPMI 1640 medium (Life Technologies, Gaithersburg, USA) containing 10% fetal bovine serum (FBS), 100 UI/ml penicillin and 100 μg/ml streptomycin (Biochrom AG, Berlin, Germany). Huh7 cells were cultured in Dulbecco's modified Eagle medium (DMEM) (Life Technologies, Gaithersburg, USA) with 10% FBS, 100 UI/ml penicillin and 100 μg/ml streptomycin. Cells were maintained at 37°C and in 5% CO_2_.

Gemcitabine (Lilly, Indianapolis, USA) and doxorubicin (Sandoz Pharmaceuticals GmbH, Holzkirchen, Germany) were dissolved in medium. The Raf1 inhibitor GW5074 and MEK inhibitor U0126 were purchased from Calbiochem (Darmstadt, Germany). MEK inhibitor AZD6244 was purchased from OTAVA (Toronto, Canada). Inhibitors were dissolved in DMSO and 0.1% DMSO (V/V) or 0.2% DMSO (V/V) was used as vehicle control.

### Cell viability test

HCC cells were seeded in 96-well-plate containing 100 μl medium at a density of 4,000 cells per well. After 48 hours incubation, cells were treated. Then the medium was discarded carefully and the cells were stained with crystal violet (0.2 g crystal violet dye, 2 ml ethanol in 98 ml distilled water) for 15 min. The crystal violet was discarded, and the wells were washed with distilled water twice and then dried. Then 200 μl straight methanol was added into each well. The optical density was read at 570 nm by Biochrom Anthos 2010 microplate reader (Biochrom LTD. Cambridge, UK). Cell viability test were done in triplicate and three independent experiments were performed. Additive effect of MEK inhibitor and chemodrugs was analysed by Bliss independence model of additivity. The fractional response to drug A alone is Fa, and the fracitonal response of drug B alone is Fb. If the total response to a mixture of the two drugs is more than Fa+Fb-Fa*Fb, it can be assumed that these two drugs are additive.

### Western blot

Cells were lysed with RIPA buffer (Sigma Aldrich, Munich, Germany) for 10 min on ice. Then the lysates were centrifuged for 15 min at 4°C with the speed of 16,100 rcf. After that, the supernatant was collected and protein concentration was determined by BCA method using BCA™ Protein Assay Kit (Thermo Scientific, Rockford, USA). ×20 μg of whole cell extracts were heated with LDS sample buffer (Invitrogen, Carlsbad, USA) at 70°C for 10 min. Then the protein was separated by SDS-PAGE in 4-12% Bis-Tris gel (Invitrogen, Carlsbad, USA) and transferred to the Pure Nitrocellulose Membrane (BIO-RAD, California, USA). After blocking for one hour, the membrane was incubated with primary antibodies at 4°C overnight. Then the membrane was probed with horseradish-peroxidase conjugated secondary antibody (Santa Cruz, Heidelberg, Germany) for one hour at room temperature. The bands were visualised by West PICO Chemiluminescent substrate (Thermo Scientific, Rockford, USA) and photographed by image acquisition system (Vilber, Eberhardzell, Germany). The band density was analysed by ImageJ and the relative expression of MRP1 and MRP3 were calibrated by the actin.

The antibodies for western blot were purchased from: Actin (A1978) (Sigma Aldrich, Steinheim, Germany); p-ERK (#4377), p-MEK (#9121), MEK (#4694), p-Raf1 (#9427), and Raf1 (#9422) (Cell Signaling, Frankfurt, Germany); MRP3 (sc-20767), ERK (sc-135900), and the secondary antibodies goat anti rabbit (sc-2004) as well as goat anti mouse (sc-2005) (Santa Cruz, Heidelberg, Germany); MRP1 (ab32574) (Abcam, Cambridge, UK).

### Intracellular doxorubicin accumulation

Intracellular doxorubicin accumulation was measured by flow cytometry (FACS) analysis. HepG2 or Huh7 cells were seeded and cultured in 10 cm plates for 48 hours. Then cells were treated with U0126 or AZD6244 for another 48 hours. After the treatment, the cells were washed with PBS, and incubated with doxorubicin (6 μg/ml) for 2 hours. Then the cells were trypsinized and resuspended in PBS followed by FACS analysis with BD FACScan System (Becton Dickinson, New Jersey, USA). The red fluorescence for doxorubicin in FL2 channel (Ex_max_ 496 nm/Em_max_ 578 nm) was used. 50, 000 cells were collected. The data was analysed by FlowJo 7.6.2 (Treestar, Ohio, USA).

### Statistics

The results were presented as mean values ± standard deviation (SD). And difference was determined by using one-way analysis of variance (ANOVA) test followed by Student-Newman-Keuls test. The statistical significance was defined as *P*<0.05. All statistical analysis was performed by SigmaStat 2.03 (Jandel Scientific, San Rafael, CA, USA).

## Abbreviations

ABC protein: ATP binding cassette proteins; EGFR: Epidermal growth factor receptor; ERK: Mitogen-activated protein kinase; MAPK: Mitogen activated protein kinase; MEK: Mitogen-activated protein kinase kinase; MRP1: Multidrug resistance-associated protein 1; MRP3: Multidrug resistance-associated protein 3; PDGFR: Platelet-derived growth factor receptor; PGP: P-glycoprotein; Raf: Proto-oncogene serine/threonine-protein kinase; siRNA: Small interfering RNA; VEGFR: Vascular endothelial growth factor receptor.

## Competing interests

The authors declare that they have no competing interests.

## Authors’ contributions

KH, PS and MB conceived and designed the study. SL and KH drafted the manuscript. SL, ZX, EM UG and NJ performed the experimental studies. All authors have read and approved the final manuscript.
